# Regulation of Ischemic Long-Term Potentiation in GluN2B and FKBP51 Underlying Cathodal Direct Current Stimulation

**DOI:** 10.1007/s11064-025-04578-6

**Published:** 2025-10-15

**Authors:** Chi-Wei Lee, Chih-Wei Tang, Ching-Hsiang Chang, Chu-Ming Chia, Tsung-Han Hsieh, Hsiang Chi, Hui-Ching Lin

**Affiliations:** 1 Department and Institute of Physiology, College of Medicine, National Yang Ming Chiao Tung University, Taipei, Taiwan; 2 Brain Research Center, National Yang Ming Chiao Tung University, Taipei, Taiwan; 3 Department of Neurology, Far Eastern Memorial Hospital, New Taipei City, Taiwan; 4 Ph.D. Program in Medical Neuroscience, College of Medical Science and Technology, Taipei Medical University, Taipei, Taiwan

**Keywords:** Ischemic long-term potentiation, FKBP51, GluN2B, Cathodal direct current stimulation, Oxygen-glucose deprivation

## Abstract

Pathological synaptic plasticity, manifested as ischemic long-term potentiation, drives neuronal hyperexcitability following oxygen-glucose deprivation. While ischemic long-term potentiation (iLTP) is mediated by excessive GluN2B-containing N-methyl-D-aspartate receptors (NMDARs) activity, the molecular mechanisms sustaining this aberrant plasticity remain poorly understood. FK506-binding protein 51 (FKBP51), a stress-related modulator of plasticity, has emerged as a potential regulator; however, its role in iLTP has not yet been explored. Cathodal direct current stimulation (cDCS), known to suppress pathological hyperexcitability, may offer therapeutic modulation of this process. In this study, we demonstrated that oxygen-glucose deprivation (OGD) induces iLTP alongside FKBP51 upregulation and GluN2B surface accumulation. Genetic deletion of FKBP51 or its pharmacological inhibition (SAFit2, 0.5 µM) normalized synaptic strength and GluN2B expression, establishing FKBP51’s critical role in maintaining iLTP. Notably, cDCS intervention replicated these protective effects, attenuating both iLTP and FKBP51 elevation. Our findings identify FKBP51 as a key mediator of ischemic synaptic dysfunction and suggest that cDCS may exert its therapeutic effects through FKBP51-dependent regulation of plasticity, thereby offering new targets for the treatment of ischemic brain injury.

**Supplementary Information:**

The online version contains supplementary material available at 10.1007/s11064-025-04578-6.

## Introduction

Stroke is the second-leading cause of disability and mortality worldwide. Current therapeutic strategies primarily focus on recanalizing the occluded vessel; however, the subsequent reperfusion of blood into the ischemic region can induce excitotoxicity, oxidative stress, and inflammation, ultimately exacerbating neuronal injury. iLTP is an abnormal form of synaptic plasticity that was initially found to be induced by pathological stimuli such as anoxia or hypoxia, through the activation of NMDAR and increased intracellular calcium ion concentration [1, 2]. Furthermore, the overexpression of NMDARs caused by glutamate accumulation leads to excitotoxicity.

FK506-binding protein 51 (FKBP51) is an immunophilin that binds to the immunosuppressants FK506 or rapamycin. It also acts as a co-chaperone of heat shock protein 90, mediating numerous cellular and transcriptional functions by regulating glucocorticoid receptor activity. FKBP51 has been linked to various disorders, including metabolic diseases, stress-related conditions, chronic pain, and neurological disorders [3, 4]. It also plays a role in psychiatric conditions such as depression, bipolar disorder, and schizophrenia through its involvement in neuronal function, synaptic plasticity, autophagy, and DNA methylation [5]. FKBP51 mRNA and protein levels are elevated in the plasma of acute ischemic stroke patients, correlating positively with the National Institute of Health Stroke Scale (NIHSS) score and the diffusion-weighted imaging (DWI) volume of the lesion. Moreover, FKBP51 levels are increased in the oxygen and glucose deprivation and reoxygenation (OGD/R) model. Overexpression of FKBP51 reduces cell viability, while silencing FKBP51 restores cell viability by regulating autophagy via the AKT/FOXO3 pathway in the OGD/R model [6]. These findings suggest that FKBP51 may play a key role in modulating synaptic plasticity in the context of stroke. We propose that FKBP51 is crucial for neuronal plasticity and disease progression during the stages of stroke.

Transcranial direct current stimulation (tDCS) is a non-invasive therapeutic technique that offers a safe and standardized method for modulating brain excitability. tDCS has been applied to various neurological conditions such as Alzheimer’s disease [7], cognitive impairment, and stroke, enhancing both motor and cognitive functions [8]. The application of tDCS has been widely studied in clinical and neuroscientific research, with stimulation delivered in opposite polarities—anodal and cathodal—leading to depolarization and hyperpolarization of the resting membrane potential, respectively [9]. In line with DCS polarity, anodal DCS enhances cortical excitability, while cathodal DCS produces inhibitory effects on neuronal activity during and for several minutes following stimulation [10, 11]. These excitatory and inhibitory effects have been used at different stages of stroke and in different injured brain regions as therapeutic strategies in clinical research [12]. DCS-induced plasticity appears to be mediated by glutamatergic synapses, particularly involving N-methyl-D-aspartate (NMDA) receptors. Pharmacological studies have demonstrated that NMDA receptor blockade prevents tDCS-induced excitability changes, while NMDA receptor agonists enhance the excitability effects induced by anodal tDCS [13]. In this study, we aimed to investigate the involvement of FKBP51 in stroke-related pathology by validating its role through genetic knockdown and chemical inhibition. Given that brain stimulation modulates stroke-related pathology, we further hypothesized that FKBP51 may contribute to the mechanisms underlying cathodal direct current stimulation (cDCS)-mediated regulation following oxygen-glucose deprivation (OGD).

In several neurological disorders, tDCS has been reported to enhance motor and cognitive function, suggesting that it modulates plasticity, neuronal morphology, and synaptic transmission via multiple pathways, including the glutamatergic system, in both animal and cell models. Similar to previous findings, tDCS has also been shown to improve working memory in stroke patients in clinical studies.

## Materials and methods

### Animals

All animal procedures were approved by the Experimental Animal Review Committee at National Yang Ming Chiao Tung University and were conducted following both the Guide for the Care and Use of Laboratory Animals and the National Institutes of Health (NIH, USA) guidelines for the care and use of experimental animals. Offspring were housed in groups of five per cage, provided with ad libitum access to food and water, and maintained in a temperature-controlled (24 °C) animal facility under a 12:12 h light/dark cycle, with lights on at 7:00 AM. Homozygous *Fkbp5* knockout mice (*Fkbp5*^tm1Dvds^, JAX stock #017989) were obtained from The Jackson Laboratory (Bar Harbor, ME, USA) and maintained on a C57BL/6J background. This conventional knockout line was generated by replacing exon 2 of the *Fkbp5* gene with a *lacZ/neo* cassette, resulting in a null allele. Previous studies had validated the lack of *Fkbp5*^−/−^ mRNA and FKBP51 protein expression in the brain of *Fkbp5* knockout mice, including the hippocampus, as confirmed by RT-PCR and Western blot [14, 15]. There is no report about abnormal developmental consequences in Fkbp5 knockout mice on The Jackson Laboratory.

### Brain Slice Preparation and Electrophysiology Recording

Animals aged 8–12 weeks were sacrificed by rapid decapitation. Their brains were removed and placed in a beaker containing cold (4 °C) oxygenated (saturated with 95% O2 and 5% CO2) artificial cerebrospinal fluid solution (ACSF) and hemisected, then cut into slices transversely (400 μm thick). Appropriate slices were then placed in another beaker containing oxygenated ACSF at room temperature for habituation. The ACSF solution had the following composition (in mM): 87 NaCl, 2.5 KCl, 0.5 CaCl2, 4 MgCl2, 23 NaHCO3, 1.25 NaH2PO4, and 25 glucose. After a habituation period of at least 1 h, the slices were transferred to a recording chamber in which they were continually perfused with room temperature, oxygenated ACSF (about 25 °C). To record the field excitatory postsynaptic potential (fEPSP) in the hippocampus, a concentric bipolar stimulating electrode (FHC, Bowdoinham, ME, USA) was placed in the CA3 region, and a capillary glass recording electrode filled with 3 M NaCl solution was placed in the CA1 region to evoke and record responses of the Schaffer collateral pathway. Baseline synaptic transmission was assessed by generating an input-output (I/O) curve using increasing stimulus intensities to evoke field excitatory postsynaptic potentials (fEPSPs) in the CA1 region. The test stimulus intensity used in subsequent recordings was set to elicit an fEPSP slope corresponding to approximately 30–50% of the maximum response. To evaluate short-term presynaptic plasticity, paired-pulse facilitation (PPR) was measured by delivering two consecutive stimuli at a fixed interpulse interval (IPI) of 30 ms, ranging from 30 to 360 ms. PPR was calculated as the ratio of the amplitude of the second fEPSP to the first. The slope of the field excitatory postsynaptic potential (fEPSP) was quantified using Clampfit (Molecular Devices) by calculating the linear portion of the rising phase between 10% and 90% of the peak amplitude, a range commonly adopted in electrophysiological studies to exclude stimulus artifacts and minimize contamination from population spikes or nonlinear components. For experiments involving pharmacological treatment, SaFit2 (0.5 µM; dissolved in DMSO and diluted into ACSF) was added to the perfusion solution.

### OGD-induced Model

Acute hippocampal slices were subjected to brief oxygen-glucose deprivation (OGD) by replacing the normal oxygenated ACSF (bubbled with 95% O₂/5% CO₂) with a modified glucose-free ACSF in which equimolar sucrose replaced glucose. This OGD solution was pre-equilibrated with 95% N₂/5% CO₂ for at least 30 min prior to perfusion to ensure complete deoxygenation [16]. The deoxygenated, glucose-deprived solution was applied for a defined period of 5 min to induce the ischemic insult [17], after which the perfusion was returned to oxygenated, glucose-containing ACSF to initiate reperfusion. The use of a 5-minute OGD protocol is supported by both published literature and our own prior findings. Brief OGD insults lasting 3–10 min have been widely reported to induce ischemic long-term potentiation (iLTP) in hippocampal slices, while preserving slice viability [10, 16, 18]. Consistent with these studies, our laboratory previously demonstrated that a 5-minute OGD using sucrose-based, N₂/CO₂-gassed ACSF reliably evokes a robust, long-lasting potentiation of field EPSPs following reoxygenation [19, 20].

### DCS Model

Two silver wires were placed in the recording chamber or plate with oxygenated ACSF, which was located on top and bottom, and a space remained for the brain slice, about 10–15 mm. Direct current stimulation voltage was applied at a current intensity of 20 V/m for 15 min. Two groups of current direction were recorded by electrophysiological recording and further analyzed by western blot assay.

### Western Blot Assay

Brain tissue which collected from hippocampus were lysised by 1% Triton X-100, 0.1% SDS, 50 mM Tris-HCl, pH 7.5, 0.3 M sucrose, 5 mM EDTA, 2 mM sodium pyrophosphate, 1 mM sodium orthovanadate, and 1 mM enylmethylsulfonyl fluoride, supplement with a complete protease inhibitor cocktail and centrifuged at 12,000 rpm for 30 min to collect the supernatants. Samples were measured by protein assay and separated by SDS-PAGE electrophoresis and transferred to a PVDF membrane. Incubation in primary antibodies overnight at 4 °C after blocking in 10% non-fat dry milk in 1XPBS for 1 h at room temperature (25–28 °C). The ECL kit was applied for immunoreactivity detection. Expression of protein will be normalized to expression of internal control, which will be measured by ImageJ.

### Statistical Analysis

All data are expressed as the mean ± SEM. The number of animals used is indicated by n. The significance of the difference between groups will be calculated by one-way ANOVA, followed by Bonferroni post hoc comparisons. Probability values (p) of less than 0.05 will represent significant differences.

## Results

### Abnormal Synaptic Plasticity Under OGD Induction was Diminished in FKBP5 KO Mice

**Fig. 1:**
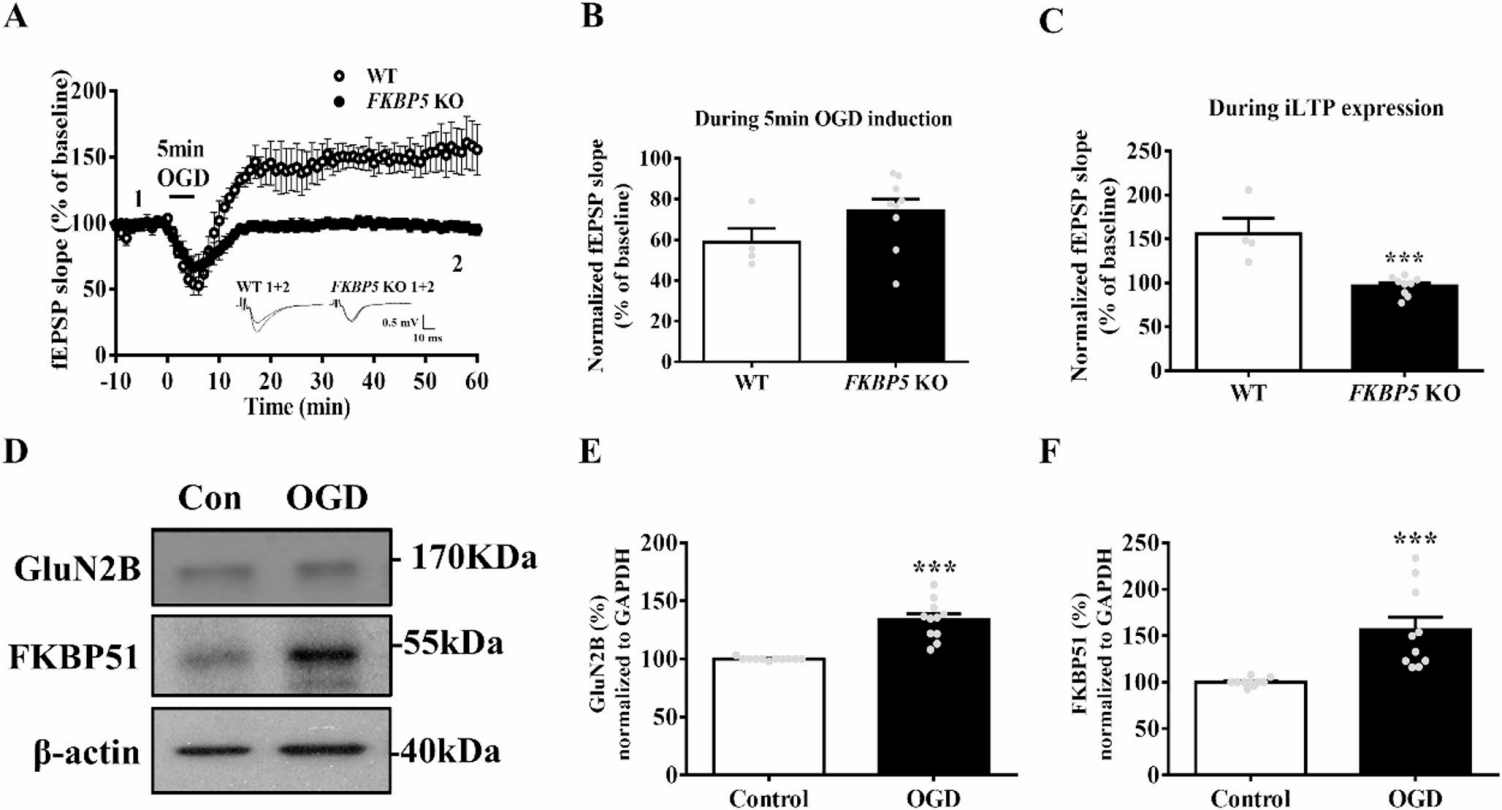
Attenuation of OGD-induced aberrant synaptic plasticity in FKBP5 KO mice. **A** Representative traces and time course of fEPSP slope recorded in the hippocampus during baseline, OGD induction, and iLTP expression over a 60-minute period **B** Summary of mean fEPSP slopes during the 5-minute OGD induction phase in WT and FKBP5 KO mice (WT, *n* = 4 slices from 3 mice; FKBP5 KO, *n* = 9 slices from 5 mice). Data are presented as mean ± SEM; Student’s t-test. **C** Summary of mean fEPSP slopes during the iLTP expression phase (last 10 min of recording) in WT and FKBP5 KO mice (WT, *n* = 4 slices from 3 mice; FKBP5 KO, *n* = 9 slices from 5 mice). Data are presented as mean ± SEM; ***p < 0.001 compared with the WT group; Student’s t-test. **D** Representative Western blots showing protein levels of GluN2B and FKBP51 in the hippocampus of control and OGD-treated groups. **E** Quantification of GluN2B expression levels (*n* = 10). Data are presented as mean ± SEM; ***p < 0.001 compared with the control group; Student’s t-test. **F** Quantification of FKBP51 expression levels, analyzed using ImageJ and normalized to the control group (*n* = 10). Data are presented as mean ± SEM; ***p < 0.001 compared with the control group; Student’s t-test

To examine the role of FKBP51 in synaptic plasticity under ischemic conditions, we evaluated changes in synaptic plasticity following OGD induction in wild-type (WT) and *FKBP5* knockout [7] mice. The fEPSP slope showed no change during the 5-minute OGD induction in either WT or *FKBP5* KO mice. However, the elevated fEPSP slope typically observed during iLTP expression was significantly reduced in *FKBP5* KO mice (Fig. 1A–C; WT, *n* = 4 slices from 3 mice; *FKBP5* KO, *n* = 9 slices from 5 mice; t-test revealed the normalized fEPSP slope during the 5-minute OGD phase: t_(11)_ = 1.535; during the final 10 min of the iLTP expression phase: t_(11)_ = 4.832, *p* < 0.0005). Expression of the NMDAR subunit GluN2B was significantly increased following OGD induction (Fig. 1E; control, *n* = 11; OGD, *n* = 11; t-test of GluN2B expression normalized to internal control: t_(20)_ = 6.684, *p* < 0.0001). Similarly, FKBP51 levels were also elevated after OGD induction (Fig. 1F; control, *n* = 10; OGD, *n* = 10; t-test of FKBP51 expression normalized to internal control: t_(18)_ = 4.019, *p* < 0.0001). To confirm that the observed potentiation following OGD represents pathological iLTP rather than canonical NMDAR-dependent LTP, we conducted pharmacological validation using the NMDA receptor antagonist D-APV (50 µM). When D-APV was continuously perfused throughout the OGD induction period, no lasting potentiation of fEPSP slope was observed, indicating that the iLTP induced by OGD is NMDAR-dependent and not attributable to nonspecific plasticity or passive recovery (Figure S1). These results align with prior findings [10], where brief OGD failed to induce iLTP in the presence of NMDAR antagonism. To further determine whether the observed molecular and electrophysiological changes were sex-specific, we repeated the OGD-iLTP experiments in hippocampal slices from females mice. Similar to the findings in males, OGD induced a robust, long-lasting potentiation of fEPSPs in female slices. Moreover, Western blot analyses revealed a significant upregulation of both GluN2B and FKBP51 expression in the OGD-treated female group compared to the control. These results indicate that the excitotoxic response and associated GluN2B-FKBP51 signaling cascade are preserved across sexes under ischemic conditions (Figure S2).

### Abnormal Synaptic Plasticity Under OGD Induction Was Blocked by the Administration of FKBP51 Inhibitor SaFit2

**Fig. 2:**
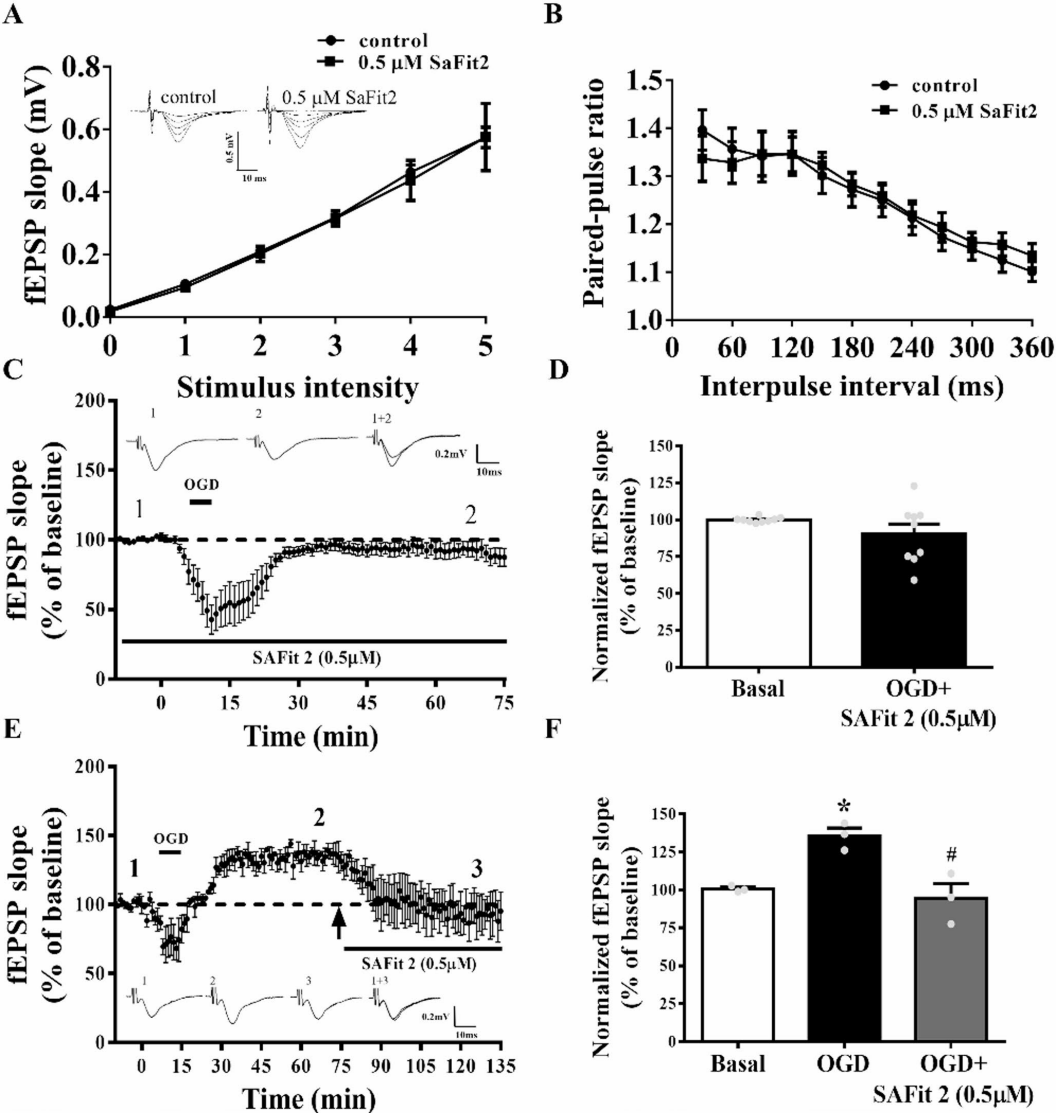
FKBP5 inhibitor SaFit2 prevents OGD-induced aberrant synaptic plasticity. **A** Representative traces and the Input-output (I–O) curve in control and 0.5 µM SaFit2 groups (*n* = 6 slices from 3 mice). Data are presented as mean ± SEM; Student’s t-test. **B** Paired-pulse ratio (PPR) evoked at a function of intervals of 30 to 360 ms in control and 0.5 µM SaFit2 groups (*n* = 7 slices from 5 mice). Data are presented as mean ± SEM; Student’s t-test. **C** Representative traces and time course of fEPSP slope recorded in the hippocampus during baseline and OGD induction over a 75-minute period. **D** Summary of mean fEPSP slopes during the 5-minute OGD induction phase in basal and OGD + 0.5 µM SaFit2 pretreatment groups (*n* = 9 slices from 4 mice). Data are presented as mean ± SEM; Student’s t-test. **E** Representative traces and time course of fEPSP slope recorded during baseline, OGD induction, and OGD + 0.5 µM SaFit2 administration. **F** Summary of mean fEPSP slopes during the 5-minute OGD induction phase in basal, OGD, and OGD + 0.5 µM SaFit2 groups (*n* = 3 slices from 3 mice). Data are presented as mean ± SEM; *p < 0.05 compared with basal, #*p* < 0.05 compared with OGD; one-way ANOVA with Bonferroni *post hoc* test

The effects of the FKBP51 inhibitor SaFit2 on abnormal synaptic plasticity under ischemic conditions were investigated. To verify whether manipulation of FKBP51 affects physiological basal neurotransmission, input-output (I/O) curves and paired-pulse ratio (PPR) were measured with application of 0.5 µM SaFit2 to hippocampal slices. The I/O curves (Fig. 2A; basal, *n* = 6 slices from 3 mice; 0.5 µM SaFit2, *n* = 6 slices from 3 mice; t-test revealed the normalized fEPSP slope of following stimulus intensity: t_(10)_ = 0.0691) and PPR (Fig. 2B; basal, *n* = 7 slices from 5 mice; 0.5 µM SaFit2, *n* = 7 slices from 5 mice; t-test revealed the normalized fEPSP slope of following interpulse interval: t_(22)_ = 0.1387) showed no significant differences between control and 0.5 µM SaFit2-treated slices, indicating that FKBP51 inhibition does not affect basal synaptic transmission or short-term presynaptic plasticity. The fEPSP slope showed no significant change after OGD induction in slices pretreated with 0.5 µM SaFit2 (Fig. 2C, D; basal, *n* = 9 slices from 4 mice; OGD + SaFit2 (0.5 µM), *n* = 9 slices from 4 mice; t-test revealed the normalized fEPSP slope during basal and OGD induction following 0.5 µM SaFit2 pretreatment: t_(16)_ = 1.426). Moreover, the increase in fEPSP slope typically observed after OGD induction was reversed by 0.5 µM SaFit2 administration, indicating that inhibition of FKBP51 can modulate synaptic plasticity under ischemic conditions (Fig. 2E, F; basal, *n* = 3 slices from 3 mice; OGD, *n* = 3 slices from 3 mice; OGD + SaFit2 (0.5 µM), *n* = 3 slices from 3 mice; one-way ANOVA revealed the normalized fEPSP slope across basal, OGD induction, and OGD with 0.5 µM SaFit2 pretreatment: F_(2, 6)_ = 12.21, *p < 0.05 compared with basal, #*p* < 0.05 compared with OGD).

### Synaptic Plasticity was Modulated by Anodal and Cathodal DCS

**Fig. 3:**
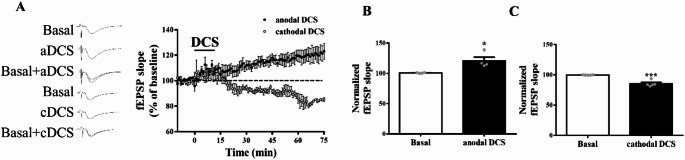
Differential modulation of synaptic responses and GluN2B/FKBP51 expression by anodal and cathodal DCS. **A** Representative traces and average time course of fEPSP slope recorded in the hippocampus during a 75-minute period with anodal or cathodal DCS intervention. **B** Summary of mean fEPSP slopes at baseline and during DCS intervention in the anodal DCS group. *n* = 4 slices from 3 mice. Data are presented as mean ± SEM in each experiment. ^*^p < 0.05 compared with basal; Student’s t-test. **C** Summary of mean fEPSP slopes at baseline and during DCS intervention in the cathodal DCS group. *n* = 5 slices from 3 mice. Data are presented as mean ± SEM in each experiment. ^***^p < 0.001 compared with basal; Student’s t-test

In the present study, anodal and cathodal DCS were applied to hippocampal brain slices to assess their effects on synaptic response modulation. We found that fEPSP slopes increased following anodal DCS and decreased following cathodal DCS (Fig. 3A–C; aDCS, *n* = 4 slices from 3 mice; cDCS, *n* = 5 slices from 3 mice; t-test revealed the normalized fEPSP slope during basal conditions and after aDCS or cDCS intervention: t_(6)_ = 3.320, *p < 0.05 for aDCS; t_(8)_ = 6.621, ***p < 0.001 for cDCS), suggesting that synaptic activity is modulated in a polarity-dependent manner by the direction of current stimulation.

### iLTP Induced by OGD was Reversed by Cathodal DCS

**Fig. 4:**
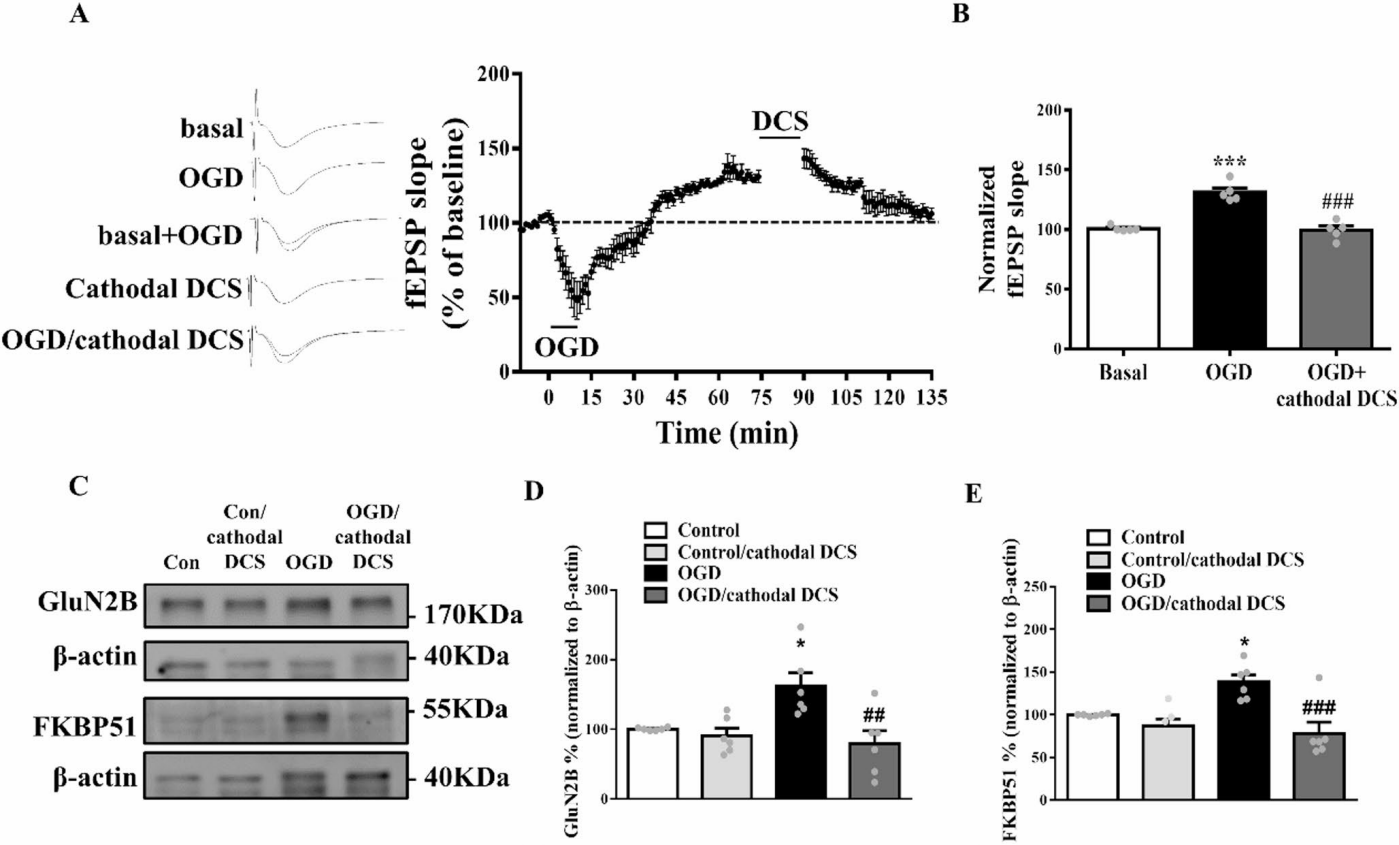
Cathodal DCS reverses OGD-induced iLTP and downregulates GluN2B and FKBP51 expression. **A** Average time course of fEPSP slope recorded in the hippocampus during a 135-minute period covering baseline, OGD induction, and cathodal DCS intervention. **B** Summary of mean fEPSP slopes at baseline, during OGD induction, and cathodal DCS intervention. *n* = 5 slices from 5 mice. Data are presented as mean ± SEM in each experiment. ^***^p < 0.001 compared with basal, ^###^*p* < 0.001 compared with OGD; one-way ANOVA with Bonferroni *post-hoc*. **C** Representative Western blots showing protein levels of GluN2B and FKBP51 in the control, control + cathodal DCS, OGD, and OGD + cathodal DCS groups. **D** Quantification of GluN2B expression levels. *n* = 6 in each group. Data are presented as mean ± SEM in each experiment. ^*^p < 0.05 compared with basal, ^##^*p* < 0.01 compared with OGD; one-way ANOVA with Bonferroni *post-hoc*. **E** Quantification of FKBP51 expression levels. *n* = 6 in each group. Data are presented as mean ± SEM; ^*^p < 0.05 compared with indicated control, ^###^*p* < 0.001 compared with OGD group; one-way ANOVA with Bonferroni *post-hoc*

Based on the findings shown in Fig. 3, we applied cathodal DCS to further investigate its therapeutic effects on ischemia-induced iLTP. The increased fEPSP slope induced by 5 min of OGD was reversed by cathodal DCS intervention (Fig. 4A–B; basal/OGD + cDCS, *n* = 5 slices from 5 mice; one-way ANOVA revealed the normalized fEPSP slope during basal, OGD induction, and OGD with cathodal DCS treatment: F_(2, 12)_ = 1.187, ***p < 0.001 compared with basal; ###*p* < 0.001 compared with OGD), indicating that cathodal DCS regulates synaptic plasticity under ischemic conditions. Supporting this, the elevated levels of GluN2B and FKBP51 observed under OGD induction were also reduced by cathodal DCS.

## Discussion

**Fig. 5:**
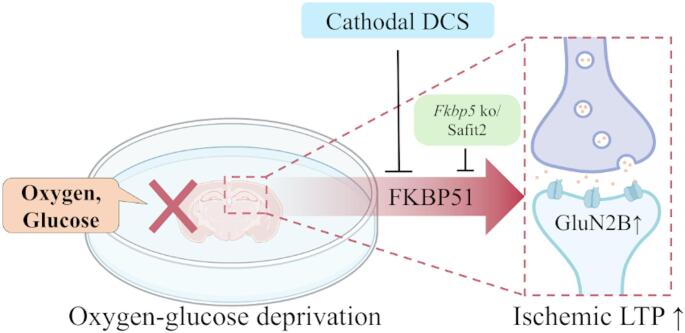
GluN2B-mediated ischemic LTP is underpinned by FKBP51, which can be inhibited by cathodal DCS. This schematic diagram illustrates the crucial role of FKBP51 in ischemic LTP and highlights the potential of cathodal DCS to target this pathological form of synaptic plasticity. Following OGD induction, ischemic LTP was observed, accompanied by elevated expression of the NMDAR subunit GluN2B and FKBP51. The OGD-induced potentiation and GluN2B upregulation were prevented by genetic deletion of *Fkbp*5 or by pharmacological inhibition of FKBP51 (SAFit2), thereby implicating *Fkbp5*/FKBP51 in this maladaptive plasticity. Moreover, cDCS reversed iLTP and reduced GluN2B and FKBP51 expression, indicating that both the ischemic LTP and FKBP51 upregulation are suppressible by cDCS in this GluN2B-dependent pathway

This study demonstrates that FKBP51 is essential for the manifestation of iLTP under OGD-induced conditions. In agreement with previous findings that ischemic LTP is mediated by NMDA receptors—particularly those containing the GluN2B subunit, which is critically involved in excitotoxic signaling [10, 21, 22]—we observed a significant upregulation of GluN2B expression following OGD induction. FKBP51 protein levels were also significantly elevated after OGD, consistent with reports of increased FKBP51 expression in stroke mouse models and in clinical samples from patients with ischemic stroke [6]. Importantly, genetic knockout of *FKBP5* abolished the OGD-induced increase in the fEPSP slope, indicating a critical role for FKBP51 in facilitating pathological synaptic potentiation. Consistent with this, pharmacological inhibition of FKBP51 with the selective antagonist SaFit2 similarly blocked the OGD-induced enhancement of synaptic responses. These findings underscore FKBP51 as a pivotal mediator of aberrant synaptic plasticity under ischemic stress. To explore potential therapeutic modulation of this maladaptive plasticity, we applied cathodal direct current stimulation (cDCS), a polarity-specific intervention known to suppress neuronal excitability. We found that cathodal DCS reversed the OGD-induced increase in fEPSP slope and significantly reduced expression levels of both GluN2B and FKBP51. These results reveal a previously uncharacterized mechanism by which cDCS regulates ischemia-induced pathological synaptic plasticity via modulation of NMDAR and FKBP51, highlighting its potential as a non-invasive neuromodulatory strategy for targeting ischemia-induced excitotoxicity. This mechanistic diagram is illustrated in Fig. 5.

Stroke involves complex pathophysiological processes across the acute, subacute, and chronic stages and is characterized by excitotoxicity, peri-infarct depolarizations, inflammation, and programmed cell death. In the acute phase, ischemia disrupts oxygen and glucose supply, impairs energy metabolism, and leads to membrane depolarization, excessive glutamate release via voltage-dependent calcium channels, reduced glutamate reuptake, and overactivation of NMDA receptors—ultimately driving neuronal excitotoxicity [7]. NMDAR subunits play differential roles in the pathophysiology of ischemia-induced excitotoxicity. Extrasynaptic GluN2B-containing NMDARs are considered triggers of glutamate-induced cell destruction, while synaptic GluN2A-containing NMDARs are generally described as neuroprotective modulators [21]. Although earlier studies reported no significant change in GluN2B mRNA following 4 min of OGD in acute hippocampal slice models [23], our findings revealed a significant increase in GluN2B protein levels in the OGD group, coinciding with changes in synaptic plasticity recorded electrophysiologically. This observation is consistent with previous studies implicating GluN2B in ischemia-induced neuronal damage [22, 24–26]. Notably, GluN2B-containing NMDARs are predominantly associated with excitotoxicity, especially when located at extrasynaptic sites; their blockade prior to ischemic onset has been shown to attenuate neuronal injury by preventing maladaptive receptor activation [27–29]. The observed GluN2B upregulation in our study further supports its role in iLTP induction and ischemia-related excitotoxic mechanisms.

FKBP5, also known as FKBP51, is a co-chaperone protein that plays a key role in regulating the stress response by modulating glucocorticoid receptor [30] sensitivity. It forms a complex with heat shock protein 90 (HSP90) and GR, reducing GR’s affinity for glucocorticoids and influencing downstream transcriptional responses. FKBP51 also regulates signaling pathways such as NF-κB, Akt, and GSK3β through its scaffolding functions—pathways closely linked with stress, metabolic syndrome, and stroke-related pathophysiology [31]. In addition, FKBP51 has been implicated in synaptic plasticity, neurodevelopment, and emotional regulation within the central nervous system. Notably, *Fkbp5* knockout mice display reduced LTP, correlating with decreased excitatory glutamatergic and enhanced GABAergic signaling, and reduced expression of NMDAR1, NMDAR2B, and AMPAR [32]. Consistent with these findings, our study demonstrated significant upregulation of FKBP51 following ischemia-mimicking OGD insult, while both genetic deletion of *FKBP5* and pharmacological inhibition using SaFit2 effectively blocked iLTP induction and reduced GluN2B expression. These results not only confirm the pathological upregulation of FKBP51 under ischemic conditions, as reported clinically [6], but also extend previous observations by directly linking FKBP51 to maladaptive synaptic potentiation mediated through NMDAR pathways. Thus, our data provide critical mechanistic insight into how FKBP51 contributes to ischemia-induced aberrant plasticity, positioning FKBP51 as a point of convergence between stress-responsive molecular cascades and stroke-related synaptic dysfunction. The upregulation of FKBP51 under OGD-induced ischemic conditions in our study aligns with clinical reports of elevated FKBP51 in stroke patients [6].

Transcranial direct current stimulation (tDCS) has been used to explore the potential therapeutic modulation of synaptic plasticity. tDCS is a non-invasive neuromodulatory technique known to bidirectionally alter cortical excitability and synaptic strength depending on stimulation polarity. It generally facilitates excitability and LTP-like plasticity changes, while cathodal stimulation tends to suppress neuronal activity and promote long-term depression (LTD)-like plasticity changes [33]. Accumulating evidence indicates that transcranial direct current stimulation (tDCS) engages NMDA receptor (NMDAR)-dependent mechanisms to mediate its effects on cortical plasticity. The pharmacological antagonism of NMDARs selectively abolishes long-lasting after-effects without affecting short-term excitability changes, highlighting the pivotal role of NMDARs in sustaining tDCS-induced synaptic modifications [34]. tDCS has been investigated in both clinical and preclinical stroke models [30, 35]. For example, atDCS is typically applied to the lesioned hemisphere in patients with subacute stroke as a form of cortical disinhibition to enhance excitability and promote recovery [36], while cathodal tDCS (ctDCS) is applied to the contralesional hemisphere to inhibit overactive areas. These targeted strategies, which are applied at specific times and locations, aim to optimize recovery by facilitating network restoration in the damaged hemisphere during stroke rehabilitation [8]. In our study, we found that anodal DCS enhanced the fEPSP slopes, whereas cathodal DCS had the opposite effect, indicating a polarity-dependent modulation of synaptic activity. Interestingly, cathodal DCS not only reversed OGD-induced iLTP but also suppressed OGD-induced upregulation of both GluN2B and FKBP51. This suggests that cathodal DCS may mitigate ischemia-induced synaptic dysfunction by targeting both NMDAR- and FKBP51-related signaling pathways. These findings are consistent with previous evidence that cathodal stimulation reduces excitotoxicity and modulates plasticity via NMDA receptor-dependent mechanisms. Considering that FKBP51 interacts with components of the NMDA signaling cascade and modulates stress-responsive plasticity, it may serve as a downstream effector or modulatory factor in tDCS-mediated long-term synaptic changes.

In conclusion, our study identifies FKBP51 as a novel mediator of ischemia-induced synaptic plasticity and a promising therapeutic target for mitigating maladaptive neuronal responses under ischemic conditions. We demonstrated that cathodal tDCS effectively attenuates iLTP while concurrently reducing FKBP51 expression, suggesting that non-invasive neuromodulation may restore synaptic homeostasis through FKBP51 regulation in the context of ischemic insult. Given the central role of NMDA receptors in ischemia-induced synaptic dysregulation and the clinical limitations of directly targeting NMDARs due to their essential physiological functions, FKBP51 emerges as a more specific and tractable downstream target. Broad NMDAR blockade indiscriminately suppresses normal excitatory neurotransmission, disrupting essential processes like learning and memory and leading to cognitive disturbances, psychotomimetic effects, and other dose-limiting side effects within a narrow therapeutic window [37]. By contrast, targeting FKBP51—a downstream mediator upregulated under ischemic conditions—enables a more precise intervention that selectively dampens pathological NMDAR-driven signaling pathways without abolishing normal receptor function. This strategy preserves normal synaptic transmission and minimizes global neurological side effects, positioning FKBP51 modulation as a safer and more specific therapeutic approach that circumvents the pitfalls of direct NMDAR inhibition in ischemic stroke. Importantly, our findings reveal that cathodal DCS not only suppresses iLTP but also downregulates both FKBP51 and GluN2B expression, indicating that FKBP51 modulation may underlie the therapeutic effects of cathodal DCS. These insights provide a mechanistic foundation for future strategies aimed at rebalancing synaptic plasticity during ischemic injury. Targeting FKBP51, whether through pharmacological inhibition or neuromodulation, may offer a promising translational approach to overcoming the limitations of current stroke therapies by shifting focus from direct receptor blockade to the modulation of intracellular signaling pathways. Collectively, our results position FKBP51 as a novel therapeutic target and highlight cathodal DCS as a viable strategy for intervention in stroke and related neurological disorders.

## Supplementary Information

Below is the link to the electronic supplementary material.

Supplementary Material 1

## Data Availability

No datasets were generated or analysed during the current study.
