# Supplementary material for: Regulation of Ischemic Long-Term Potentiation in GluN2B and FKBP51 Underlying Cathodal Direct Current Stimulation
Source: Neurochem Res. 2025 Oct 15;50(5):328. doi: 10.1007/s11064-025-04578-6 (PMC12528262; doi:10.1007/s11064-025-04578-6)
Supplement: Supplementary file 1 — Supplementary Material 1 [file 11064_2025_4578_MOESM1_ESM.doc]

**Regulation of Ischemic Long-Term Potentiation in GluN2B and FKBP51 Underlying Cathodal Direct Current Stimulation**

Chi-Wei Lee^1^, ^2^, **^†^**, Chih-Wei Tang^3^, Ching-Hsiang Chang^1^, Chu-Ming Chia^1^, Tsung-Han Hsieh ^1^, Hsiang Chi ^1^, Hui-Ching Lin^1,2^,*

^1^Department and Institute of Physiology, College of Medicine, National Yang Ming Chiao Tung University, Taipei, Taiwan

^2^Brain Research Center, National Yang Ming Chiao Tung University, Taipei, Taiwan.

^3^Department of Neurology, Far Eastern Memorial Hospital, New Taipei City, Taiwan

^4^Institute of Brain Science, National Yang Ming Chiao Tung University, Taipei, Taiwan

*Address for reprint requests and correspondence:

***Hui-Ching Lin* PhD*,***

Department of Physiology, College of Medicine, National Yang Ming Chiao Tung University, Taipei 11221, Taiwan; Phone: +886-2-2826-7944; Fax: +886-2-2826-4049.

E-mail: hclin7@nycu.edu.tw; [huiching4732@gmail.com](mailto:huiching4732@gmail.com)

**Acknowledgement**

We would like to thank Editage for editing the English in this manuscript and all of the research participants. We also appreciate Dr. Yi-Hsuan Lee for generously providing the *Fkbp5* knockout mice.

**Supplemental Results**

**
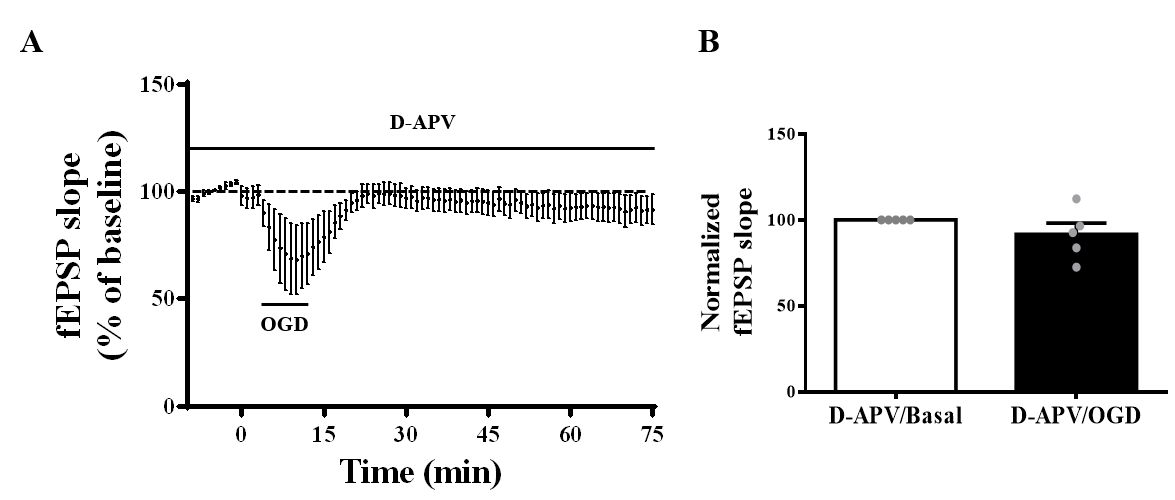
**

**Supplemental Figure 1.**

Fig. S1. Administration of NMDA receptor antagonist D-(-)-2-Amino-5-phosphonopentanoic acid (D-AP5) blocks OGD-induced iLTP.

(A) Representative traces and time course of fEPSP slope recorded in the hippocampus during baseline, OGD induction, and iLTP expression over a 60-minute period under continuous D-APV (50 μM) perfusion (*n* = 5 slices from 3 mice). D-AP5 (50 μM) was applied in ACSF and OGD solution and perfused continuously throughout the OGD induction period. (B) Summary of mean fEPSP slopes during the iLTP expression phase (last 10 minutes of recording) in the D-APV group (*n* = 5 slices from 3 mice). Data are presented as mean ± SEM; Student’s t-test.

**
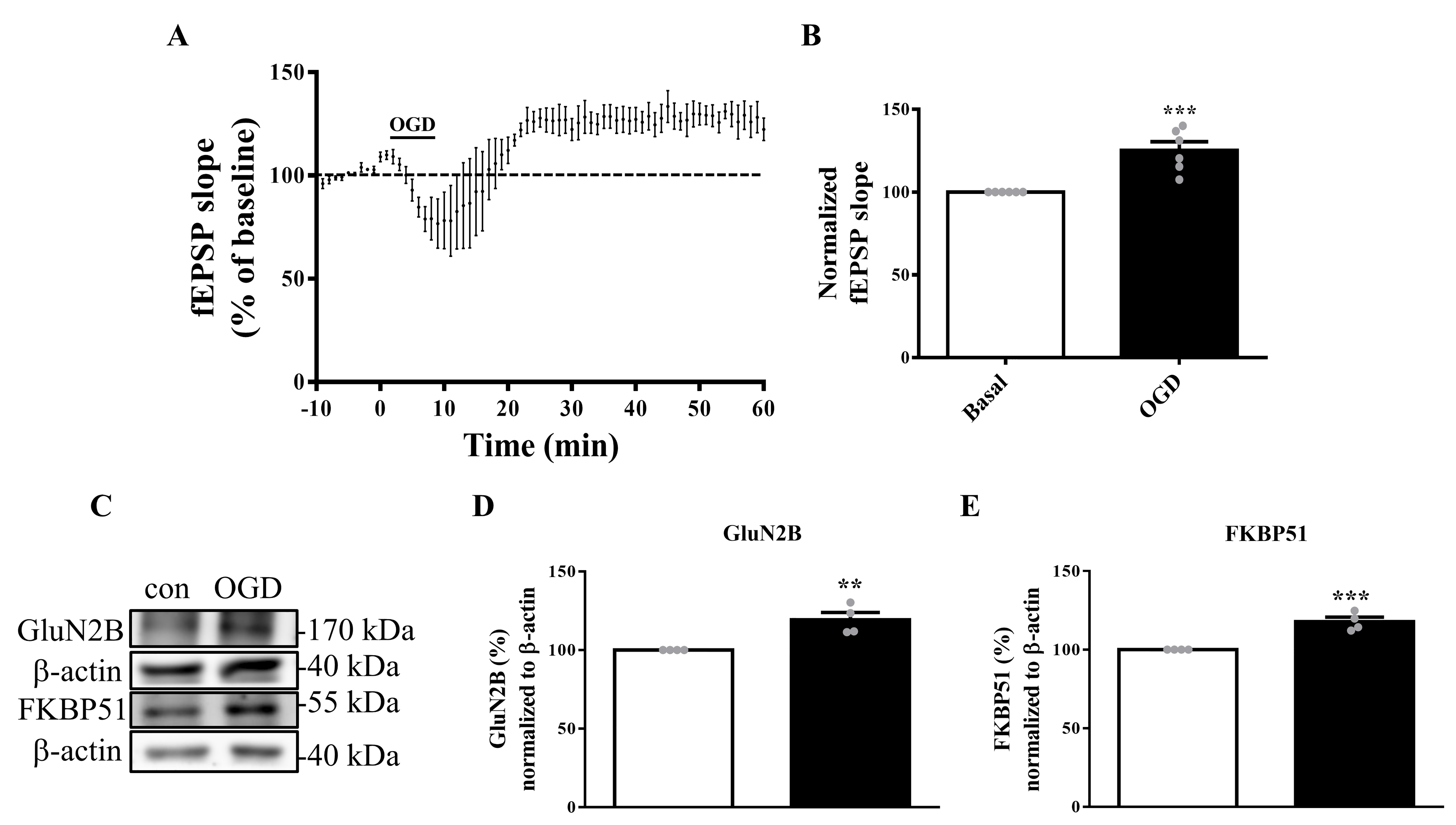
**

**Supplemental Figure 2.**

Fig. S2. Attenuation of OGD-induced aberrant synaptic plasticity in FKBP5 KO mice

(A) Representative traces and time course of fEPSP slope recorded in the hippocampus during baseline, OGD induction, and iLTP expression over a 60-minute period. (B) Summary of mean fEPSP slopes during the iLTP expression phase (last 10 minutes of recording) in female WT mice (*n* = 6 slices from 4 mice). Data are presented as mean ± SEM; ****p* < 0.001 compared with the control group; Student’s t-test. (C) Representative Western blots showing protein levels of GluN2B and FKBP51 in the hippocampus of control and OGD-treated groups. (D) Quantification of GluN2B expression levels (*n* = 4 per group). Data are presented as mean ± SEM; ***p* < 0.01 compared with the control group; Student’s t-test. (E) Quantification of FKBP51 expression levels, analyzed using ImageJ and normalized to the control group (*n* =4 per group). Data are presented as mean ± SEM; ***p* < 0.01 compared with the control group; Student’s t-test.
